# Graphene-Based Materials for Biosensors: A Review

**DOI:** 10.3390/s17102161

**Published:** 2017-09-21

**Authors:** Phitsini Suvarnaphaet, Suejit Pechprasarn

**Affiliations:** Faculty of Biomedical Engineering, Rangsit University, Pathum Thani 12000, Thailand; phitsini.suv@gmail.com

**Keywords:** graphene, functionalized graphene, biosensor devices, instrumentation, surface plasmon resonance, field-effect transistor, electrochemical, bioimaging

## Abstract

The advantages conferred by the physical, optical and electrochemical properties of graphene-based nanomaterials have contributed to the current variety of ultrasensitive and selective biosensor devices. In this review, we present the points of view on the intrinsic properties of graphene and its surface engineering concerned with the transduction mechanisms in biosensing applications. We explain practical synthesis techniques along with prospective properties of the graphene-based materials, which include the pristine graphene and functionalized graphene (i.e., graphene oxide (GO), reduced graphene oxide (RGO) and graphene quantum dot (GQD). The biosensing mechanisms based on the utilization of the charge interactions with biomolecules and/or nanoparticle interactions and sensing platforms are also discussed, and the importance of surface functionalization in recent up-to-date biosensors for biological and medical applications.

## 1. Introduction

**Figure 1:**
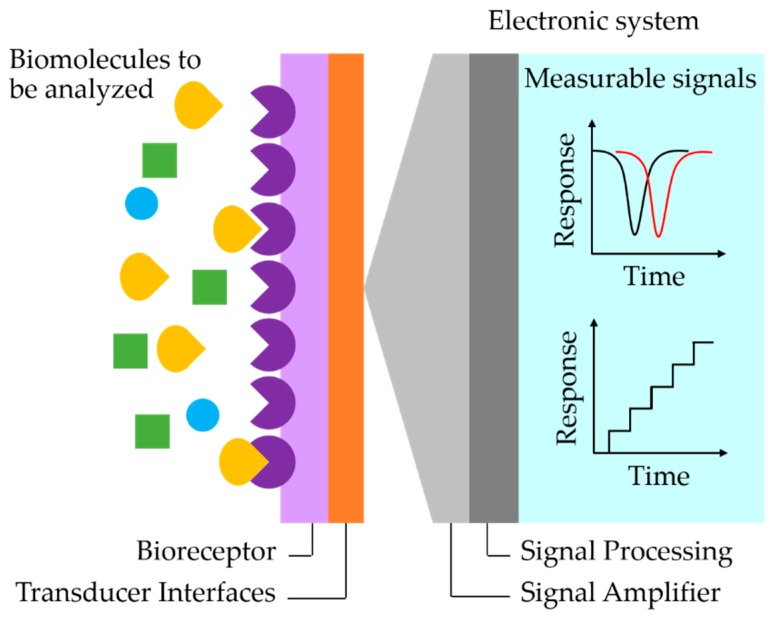
Schematic illustration of a typical biosensor system.

A biosensor is an analytical device which can detect a biomolecule-related element with an appropriate transducer to generate a measurable signal from the sample [1,2]. The biosensor system illustrated in Figure 1 shows a typical platform, consisting of a bioreceptor interfaced with a transducer. The bioreceptor has to be capable of recognizing the biomolecule element, for instance, enzymes, antibodies, DNA, RNA, and cells. In an incorporation, the biological signal that can be detected in various quantities by the receptor is transduced by physical, chemical, optical, thermal or electrochemical actions into observable information and analyzed quantitatively. The first generation of biosensor devices was introduced by Clark and Lyons [3] to monitor chemical components in the blood of a surgical patient and to record quantitative biomolecule contents in blood. Since then, the applications of biosensors in the biomedical field and global healthcare have become indispensable for human life improvements in order to spatially analyze diseases through a patient [4,5,6], to detect and to diagnose biomolecules [7,8,9,10,11], to integrate with drug delivery [12,13,14] and food safety [15]. In many efforts of the investigations, major requirements of biosensors are that the receptor has to not only be highly selective and specific to the biomolecular element, but also that the transducer needs to be ultrasensitive and with sufficient reproducibility for reliable real-time measurements. For more precision, the presence of chemical binding or biological specificity to an analyte in a labeled technique is used to ensure that only the labeled biological activities give a strong signal. However, this technique requires a labeling process in preparation and involves the fluorescent dyes, chemiluminescent molecules, photoluminescent nanoparticles and quantum dots [16,17,18,19,20,21,22,23,24]. Alternatively, the label-free technique uses molecular, physical, mechanical, electrical, optical properties and charge interaction to monitor binding activities. The label-free methods can provide real-time tracking in biomolecular events and give more direct information about the target biomolecules without interference effects from the labeling procedure. Currently, label-free biosensors are essential for personalized genomics, cancer diagnostics, and drug development [10,13,25] where the sensitivity is one of the key requirements that needs to be engineered for state-of-the-art biosensors [26].

The emergence of graphene, a two-dimensional (2D) nanomaterial, has played a tremendous role in the electronic and sensor communities. Graphene is defined as “a single-atom-thick sheet of hexagonally arranged, sp^2^-bonded carbon atoms occurring within a carbon material structure” [27]. The nano-thickness graphene film with 100 μm of lateral size is observed as carbon planes connected together by van der Waals forces acting over a distance of about 0.335 nm. The properties of graphene are those of a semi-metal and are stable under ambient circumstances. This contradicts the general belief that a 2D material could not exist and be thermodynamically stable. The charge transport and electronic properties of graphene are due to its unique electronic band structure. In particular, among existing nanomaterials graphene has a large surface area (2630 m^2^/g) [28,29] being available for direct interaction in a wide range of biomolecules [26]. Graphene can be engineered with structural defects using low-cost fabrication methods due to the migration of heteroatoms, oxidation, and reduction by chemical modification [30,31].

The uses of graphene-based materials for biosensing involve two points of view. One is based on charge-biomolecule interactions at π-π domains, electrostatic forces and charge exchange leading to electrical variations in the pristine graphene. The other uses are the effect of defects, disorder, the chemical functionalization to immobilize the molecular receptors onto the surface of graphene oxide (GO), reduced graphene oxide (RGO) and graphene-based quantum dots (GQDs). Recently, several excellent reviews have focused on the interactions of graphene, GO and RGO-based biosensors with their biomolecular targets [26,32,33,34]. In this review, we focus on the graphene-based material properties due to their fabrication process, surface chemistry and the photoluminescent graphene so-called GQDs. These graphene properties are utilized and integrated in biosensors for biological and medical applications.

## 2. Graphene-Based Materials: Fabrication Process and Properties

**Figure 2:**
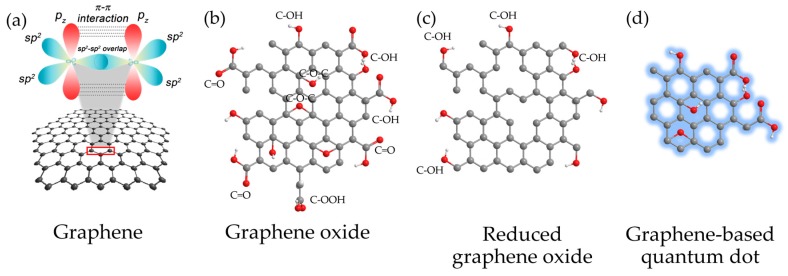
Structures of graphene-based materials show (**a**) the pristine graphene (pure-arranged carbon atoms) with sp^2^-hybridized carbon atoms, and the chemically modified graphene, including (**b**) graphene oxide (GO); (**c**) reduced graphene oxide (RGO) and (**d**) graphene quantum dot (GQD).

The synthesis of graphene-based materials in a presence of different methods can be controlled to confer properties for specific and desirable applications. Figure 2 shows the major types of graphene-based materials having been useful for engineering biosensors. The structure of pristine graphene is characterized as the array of 2D and sp^2^-hybridization of pure carbon atoms arranged in a hexagonal lattice with covalent bonds. Meanwhile, functionalized graphenes are achieved by synthesis and preparation, for examples, the carbon core structure can be oxidized forming GO, the reduced structure with vacancy defects is RGO, structures a few nanometers in size with quantum phenomena are GQDs. Interestingly, many types of the GQDs have attracted interest due to their outstanding optical characteristics in the presence of photoluminescence involving specific binding capability to a wide range of biomolecules. Such morphological and intrinsic characteristics should serve the analytical transduction of biosensors on the limit of detection (LOD), sensitivity, selectivity, repeatability, and biocompatibility.

To provide an understanding of the physics, chemistry, and engineering of graphene-based structures, and how these methods have crucial influences on their characteristic and properties, we will first discuss how graphene and related materials are prepared and synthesized.

### 2.1. Pristine Graphene

**Figure 3:**
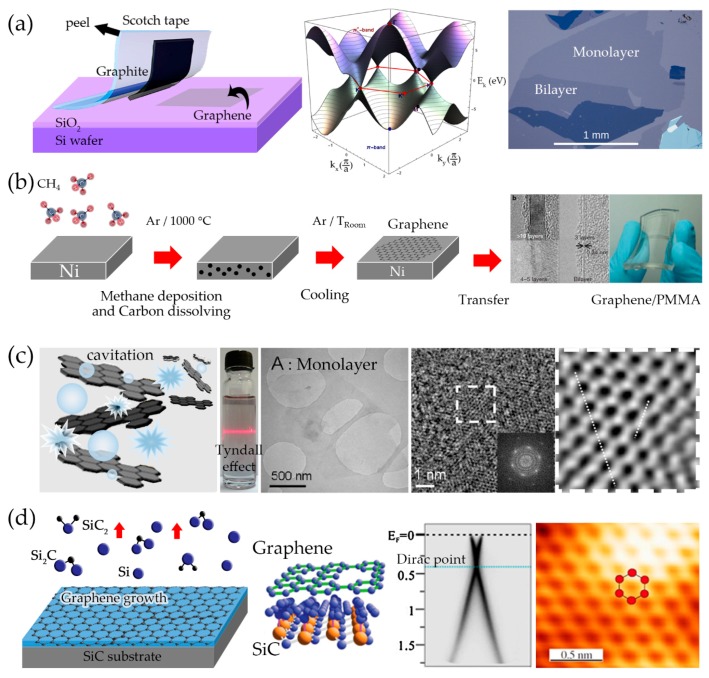
Several techniques of graphene synthesis: (**a**) graphene sheet is left on top of a silicon oxide wafer exfoliated by scotch-tape technique, its electronic band structure, and the real monolayer and bilayer graphene (Reprinted with permission from [39]); (**b**) Large scale process of graphene growth using (chemical vapor deposition) CVD and the transferred graphene to poly(methyl methacrylate) (PMMA) (reprinted with permission from [40]); (**c**) Liquid exfoliation of graphene showing crystalline honeycomb pattern on the exfoliated layer (reprinted with permission from [41]); and (**d**) epitaxial graphene growth on a silicon carbide (SiC) by sublimation of Si atoms and the structural characteristic of the monolayer graphene (reprinted with permission from [42]).

A monocrystalline graphitic film composed of a single one-atom-thick carbon layer can be prepared by peeling it from a small platelet of highly oriented pyrolytic graphite (HOPG) in a mechanical exfoliation approach with a Scotch tape, as seen in Figure 3a. Mechanical exfoliation is the technique by which a longitudinal or transverse stress is exerted on the surface to overcome the van der Waals binding energy of approximately 2 eV/nm^2^ [35]. When the graphene layer is fabricated as the conducting channel in a field-effect transistor (FET) device contacted with any metal conductor such as gold or silver, the unusual electronic properties of the graphene field-effect transistor (GFET) depend on the charge transport and electron conduction in the band structure and linear behavior at low energy, so the electrons in graphene are transported ballistically analogous to the relativistic massless fermions with an effective velocity of approximately 10^6^ m/s. The GFET can even be retained under ambient circumstances without any protection of the graphene. The charge carriers exhibit ultra-high mobility, found to be 200,000 cm^2^/Vs, at temperatures between 10–100 K, which can be induced by applying gate voltage, a strong ambipolar electric field effects in charge carrier concentrations at the range of 10^13^ cm^−2^ with a long mean free path of 400 nm, high thermal conductivity, very low resistivity (less than that of silver at room temperature), low spin-orbit coupling, high mechanical strength (200 times greater than stainless steel), high elasticity, transparent (98% transmittance) and biocompatibility [36,37,38]. As a novelty of graphene, a variety of practical applications now appear possible, and graphene and new graphene-like 2D nanomaterials are used in the manufacture of innovative electronic devices. The efficiency of graphene-based sensors and biosensors are predicted to be substantially faster than that of semiconductor-based devices resulting in more efficient computers.

However, to obtain graphene by the Scotch-tape method, the maximum dimensions of the pristine graphene that can be isolated for fundamental studies and basic research that require high quality at only 1 mm^2^. Several synthetic procedures have been established that possess scalability in their different characteristics and are more effective for matching applications.

#### 2.1.1. Chemical Vapor Deposition

Chemical vapor deposition (CVD) is one of the most popular methods for industrial-scale fabrication of graphene at this moment. Even though graphene provided by this method is very high quality and the number of layers can be precisely controlled, the electronic and structural properties has remained poorer than those of the Scotch-tape graphene. Excellent applications of CVD graphene have been performed in many fields, including electronic transistors [43,44], transparent conductive electrodes [45,46], corrosion-inhibiting coatings [47,48] and high sensitive sensors [44,49,50]. CVD is a way to dissolve a gaseous reactant, for example, methane, ethane or propane, into a heated substrate such as Cu or Ni, where the reaction results in graphene film growth on top of the substrate from the nucleation sites during the cooling process as seen in Figure 3b. A large-scale area of single-layer to few-layer graphene films is usually grown on various metal substrates such as Cu, Ni, Pt, Co, Ir and Ru [29,40,51,52,53]. To gain better control of the growth of monolayer or bilayer graphene, Ni and Cu are usually used instead of the other metals which are much higher in cost. The transferred substrate can be either a polymer or metal [53] where the transfer process may causes damages from tearing and the formation of wrinkles. It is reported that the production of transparent, conductive, and large-area graphene films was achieved by growth on Cu foil by plasma CVD at low temperature of 300–400 °C and transferred to a diagonal width up to 30 inches via roll-to-roll process. The as-prepared graphene films consist of a few-layer sheets with the transparency 97.4% relative to low sheet resistance of 125 Ω/sq which is used in touch screen displays by the touch panel screen industry [52]. Alternatively, large-size CVD graphene is introduced into possible biosensor platforms to support Au nanoparticles and then to construct biomolecule receptor patterns [54]. Graphene is presented in order to enhance the sensitivity of sensors and biosensors whereas the nanoparticles increase the surface area to bind the analyte. In a glucose or glutamate molecule biosensor [55], the glucose oxidase or glutamic dehydrogenase is functionalized on the graphene surface to monitor the conductance changes in real time. The CVD graphene is usually fabricated as a conducting layer in sensors and biosensors that require a high quality surface platform.

#### 2.1.2. Liquid Exfoliation

Liquid exfoliation is a low-cost top-down process for fabricating graphene in solution using an ultrasonic energy source to generate microcavitations and to blast the raw bulk graphite into smaller fragments and thinner layers of graphene. A number of hours are needed to effectively extract the graphite into individual layers. The approach of the liquid exfoliation is carried out in organic solvents such as *N*-methylpyrrolidone (NMP), *N*,*N*-dimethylacetamide (DMA), γ-butyrolactone (GBL) and 1,3-dimethyl-2-imidazolidinone (DMEU) [41,56,57,58,59]. Recently, studies on the exfoliation and dispersion of graphite have been carried in water/surfactant solutions instead of those organic solvents and their stabilization has been described using DLVO and Hamaker theory [41]. The dispersed graphene layers are not supposed to reaggregate due to the coated surfactant which protects against the Coulomb repulsion. The surfactant-coated exfoliated graphene can be stabilized as a colloid. The bounded-molecule surface forms a tail-group in surfactant, a so-called double layer. The possibility of exfoliation arises because of the competition (leading to an energy balance) between the interlayer van der Waals energy and the repulsive solvent-graphene interaction when the surface energy of the solvent is of matched magnitude with that of the dispersed graphene. Figure 3c shows the blasted and exfoliated dispersion of graphite prepared through ultrasonication and mild centrifugation. An individual single-layer graphene from the solution is observed in the transmission electron micrograph, where the selected electron diffraction area shows the characteristic hexagonal rings. This method is very popular due to the simple intercalation, cavitation and exfoliation procedure. The exfoliated graphite layers show high throughput during the production stage. However, prolonged ultrasonication treatment can possibly damage the exfoliated graphene sheets resulting in small size and nano-impurities. The thickness of the exfoliated graphite layers is on the order of 10 nm to 100 nm where they lose the distinctive graphene electronic properties. To improve the properties, microwave radiation and annealing have been used post-exfoliation in the removal of the trapped solvent and air bubbles, significantly expanding the exfoliated graphite layer volume and reducing the nanoscrolling effect. The use of graphene in liquid exfoliation serves as an important benchmark in the development of cost-effective graphene-based transparent electrodes and sensors with high surface area and electrocatalytic activity, optical limiters and mechanical reinforcements for polymer-based composites [59].

#### 2.1.3. Epitaxial Growth on SiC

A different method starts with silicon carbide (SiC) heated up to 1250–1450 °C under ultrahigh vacuum (UHV) as seen in Figure 3d. The silicon ions will then sublimate, leaving behind the carbon atoms on the surface of SiC. Upon cooling under optimized conditions, the carbon atoms will self-organize into a honeycomb structure. The epitaxial graphene (EG) on SiC can then be transferred onto a desired substrate as free-standing graphene. For example, EG achieves the transfer by using a thin gold layer and polyimide (PI) to peel-off from SiC to a SiO_2_/Si wafer. The resulting graphene layer however, has high amounts of defects with very low mobility of 100 cm^2^/Vs [60]. This method has difficulty in controlling the number of layers formed in the temperature control and the presence of defects or disorder in the hexagonal structure. More importantly, the electronic properties of EG have been characterized and indicate an opening of a band gap about 260 meV where the Fermi level shifts to 400 meV [61,62]. The size of the band gap also depends on the graphene thickness. A number of researchers have studied intensively the energy bandgap formation, growth mechanism, and the symmetry breaking at the graphene-SiC interface [63,64]. There are two possible origins to describe the gap opening phenomenon between the two bands. One is to hybridize the electronic states at the Dirac points, which requires the translation symmetry to be broken [65]. The other one is to break the equivalence between atomic sublattices in graphene, which does not require any translation symmetry breaking [66,67]. The opening of the gap is believed to be due to the breaking of the symmetry between the K and K’ points in the Brillouin zone by the graphene layer and the SiC substrate interaction. The EG appeared on the surface of SiC substrate which has a quasiperiodic 6 × 6 domain pattern and the atomic pattern of a $6\sqrt{3}\times 6\sqrt{3}R30^{\circ }$ periodicity [68]. The utilization of EG on SiC is promising for making an electroanalytical platform in a wide range of biosensors which is comparable to carbon nanotubes, boron-doped diamond, and glassy carbon. EG-based biosensors are demonstrated to resolve all four nucleic acid bases and are able to distinguish dopamine, ascorbic acid, and uric acid at physiological pH in which the electrochemical performances contribute to the presence of edge and plane defects [69]. Although the cost of SiC wafer remains a limiting factor for industrial fabrication, EG offers easy integration into existing electronic procedures and importantly, the energy band gap enables an electronic off-state used in graphene transistors and sensor devices.

### 2.2. Functionalized Graphene

**Figure 4:**
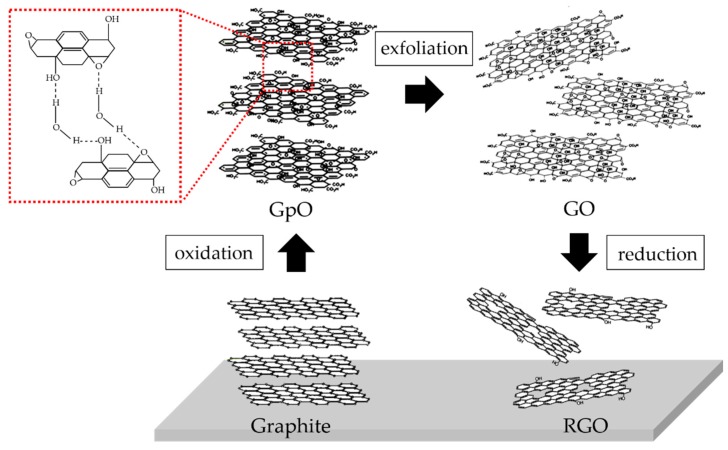
Schematic structure of chemical synthesis based on Lerf-Klinowski model.

Chemical synthesis is a cost-effective top-down nanotechnique which utilizes oxidation and reduction reactions to indirectly synthesize graphene. In fact, chemical synthesis was the first method to yield a single layer hexagonal carbon structure, but the existence of a 2D layer has not been reported. In 2004, the experimental breakthrough of graphene allowed exploration of the evidence of its extraordinary properties. Since then, the chemical method has come back to be realized in large throughput synthesis. Graphene can be facilitated via the process involving oxidation of fine graphite to graphite oxide (GpO), which is then exfoliated and reduced (chemically or thermally) or irradiated to RGO. Figure 4 shows the chemical synthesis process of graphene from raw graphite material, where the degree of elemental ratios vary based on the different chemical reaction protocols. This directly affects the stability of the chemically derived graphene and the immobilized interaction with biomolecules. Several major methods and structural models are discussed in the next section.

#### 2.2.1. Chemical Synthesis of Graphite Oxide and Graphene Oxide

There are three original methods used to synthesize graphene, including:

(1) Brodie method (1859) [70], the GpO was prepared using Ceylon as a raw material resulting in a purification to give 99.96% carbon. A boiled mixture of concentrated nitric and sulfuric acids called carbonic acid was used as an oxidizing agent. As observed from elemental analysis, the oxidized Ceylon graphite included C:O:H contents as 67.79:30.37:1.84 and the C-to-O ratio was 2.23. Therefore, the material was termed graphic acid, and was the very first sample of graphite oxide prepared experimentally.
(2) Staudenmaier method (1898) [71], this method is very similar to Brodie’s. The graphite oxide is prepared in a mixture of concentrated sulfuric acid and fuming nitric acid. In addition, potassium chlorate oxidizing agent is also added and reacted over 4 days. By rinsing in water and dispersing in diluted hydrochloric acid, sulfonate ions were removed. Finally, the graphite oxide was dried at 60 °C for 2 days. The graphite oxide prepared by this method was found to have an elemental composition of C:O:H of 58.73:23.28:17.99. The C-to-O ratio was 2.52, which indicated the lowest degree of oxidation;
(3) The Hummers and Offeman method (1958) [30] was developed when it was realized that the usage of nitric acid requires a lot of time for oxidizing graphite, has the potential for explosion and the release of highly corrosive vapor. The Hummers and Offeman method is a less hazardous way to oxidize graphite. The oxidizing agent is a mixture of concentrated sulfuric acid, sodium nitrate and potassium permanganate. The entire process needs 1–2 h to complete the reaction. As a result, the graphitic oxide had a C-to-O ratio between 2.1–2.9. The color of the product in aqueous solution is referred to the degree of oxidation. The product gives a bright yellow color for the most oxidized graphite while the green to black color refers to poor graphitic oxidation having too high C-to-O ratios. Currently, the Hummers and Offeman method is the most commonly used and is commonly known as the Hummers method.

As a result of the oxidation of graphite there is an increase in the interlayer spacing. This means that the separation between graphene interlayers is expanded by the intercalation of oxidized contents or the polar liquids such as sodium hydroxide [72] as seen in solid-state ^13^C-NMR [73]. The interlayer distance of 0.335 nm in pristine graphite increases to 0.562 nm after 1 h of oxidation and increases further to 0.737 nm after 24 h of oxidation. It is suggested that the carboxyl and alkyl groups are mostly at the edges of the GpO layers. By treatment via mechanical stirring or ultrasonication, the GpO can be exfoliated to GO easily with the same oxide contents. Moreover, the Hummers method can be improved by making a small modification [74]. The amount of potassium permanganate was doubled and the sodium nitrate was replaced by phosphoric acid in a proportion to sulfuric acid of (1:9). The improved method leads to a large amount of hydrophilic GpO being made compared to the original Hummers method. It highlights the fact that the oxidizing agents interact more with the basal planes of graphite. A high quality of GpO would promote the electrical conductivity after it was converted to graphene. Many findings make it possible to utilize GO and further composites [75,76].

Understanding of the GpO structure is needed in order to develop modifications which would improve the mechanisms in any applications. Primarily, the GpO is composed of C, O and H atoms where the C-to-O ratio for complete oxidation is in a range of 1.5–2.5 [31]. To model the GpO structure, many research groups have debated and determined the precise structure using complex and advance techniques. Seven proposed structures have been presented [77,78,79,80]. The first model was proposed by Hofmann and Hoist in 1939 [81]. The basal plane of graphite consists of 1,2-epoxide (C-O-C) groups across the sp^2^-hybridized carbon structure. The molecular structure is formulated as C_2_O. In 1947, Ruess [82] proposed a second model made up of a sp^3^-hybridized carbon structure as the basal plane whose surface is decorated with the hydroxyl (C-OH) groups. 1,3-Epoxides were placed at 1/4th of the cyclohexanes. Scholz and Boehm [83] suggested their model in 1969 where the epoxide and ether groups are absent in the carbon structure. They were replaced by ketone and hydroxyl groups by using regular quinoidal bases. In 1988, Nakajima and Matsuo [84] proposed a structural model which was similar to that of the 2-type graphite fluoride (C_2_F)_n_. The model showed hydroxylated graphite intercalation along the c-axis. There were carbonyl (C=O) and hydroxyl (C-OH) groups which were bonded to the basal carbon structure. The molecular formula was intermediate between C_8_(OH)_4_ and C_8_O_2_ with C_0_ = 8.22 × 2 Å and C_0_ = 5.52 × 2 Å, respectively. The increase in the dehydration was influential in decreasing the distance along the c-axis. This model was deduced from the elemental analysis, X-ray diffraction and chemical reactivity studies. The most well-known and widely used model is the one proposed by Lerf and Klinowski in 1996 [77,79]. Their model was derived from the ^13^C and ^1^H magnetic-angle spinning nuclear magnetic resonance (NMR) data of GpO. The evidence suggested aliphatic 6-membered rings containing hydroxyl (C-OH), epoxide (C-O-C) and double-bonded carbon (>C=C<) which indicated a basal plane structure made up of aromatic islands. The basal carbon structure was not only separated by the C-OH attachments leading to wrapping, but the graphite oxide structure was reconfigured to be tetrahedral. This leads to the strong interaction with water molecules. In this model, the 1,2 epoxide and hydroxyl groups are at the basal plane while the carboxyl and hydroxyl groups are mostly at the edge of the plane. Observations [85] supported the model of Lerf and Klinowski, where (1) the sp^2^ hybridized structure is found as a core of the graphite oxide sheet; (2) holes, a kind of defect, are present in the structure; (3) C-OH, 1,2-C-O-C groups are doped onto the structure; (4) the C=O groups are found along the edge of the holes; and (5) the oxidized regions are randomly located and no superstructures are formed. Another model was proposed by Dékány [86] as recently as 2006. X-ray photoemission spectroscopy (XPS), diffused reflectance infrared Fourier transform spectroscopy (FTIR), electron spin resonance (ESR), TEM, XRD and ^13^C-MAS NMR were used to reveal the structural details. The Dékány model was built upon the Ruess and Scholz-Boehm models. The graphite oxide contained *trans*-linked cyclohexyl chairs, having 1,3-epoxides and tertiary hydroxyls, and corrugated networks of keto/quinoidal groups. In addition, phenolic groups were inserted into this model to explain the acidity of graphite oxide. Two years later in 2008, Ruoff and colleagues [87] synthesized and determined the structural characteristics of ^13^C-labelled graphite oxide. Using ^13^C-MAS NMR analysis, hydroxyl and epoxide groups were found bonded to the carbon structure. Also, the carbonyl groups were found mostly bonded to the edge of defects. The carbon structure had non-protonated functionality as detected near 100 ppm. This was narrowed down to the possible structure of graphite detected by NMR. In 2009, Gao and Ajayan [80] offered new insights into the graphite oxide structure. The Ajayan model suggested that 5- and 6-membered-ring lactols at a level of 100 ppm were detected in the ^13^C-MAS NMR signals. The functional groups on graphite oxide included the 115 (hydroxyl and epoxide), the 3 (lactol, O-C-O), the 63 (sp^2^ hybridized carbon), the 10 (lactol:ester:acid carbonyl) and 9 (ketone carbonyl). Moreover, the GpO and GO are seen to have the acidic properties due to the ketones at the edge of carbon plane in a presence of incompletely hydrolyzed covalent sulphates, containing salts or esters of sulfuric acid. In contrast to that of usual acid properties, it originates from the presence of carboxyl groups. In fact, the acidic functional groups, i.e., vinylogous acid, on GpO and GO are originated gradually due to the interaction with water. This points out the instability of the oxide functional groups in the presence of water, leading to constantly evolving GpO and GO structures. Therefore the latest model has been proposed in terms of the dynamic-like structure.

#### 2.2.2. Reduction of Graphene Oxide

The chemical synthesis brings the opportunity to produce graphene at a mass-scale level from GpO or GO in solution. The GpO is usually exfoliated via mechanical stirring or ultrasonication. Ultrasonication is an effective and fast exfoliation method, but it may break apart and tear off the GO sheets into small fragments. In the chemical reduction procedure [88], the GO is dispersed in water in a proportion of GO to water of 100 mg to 100 mL and then treated by hydrazine hydrate (N_2_H_4_) at 1.0 mL (Mw 32.1 mmol). The reduction was carried out at 100 °C through a one-step refluxing process overnight. After the reduction, the GO turns into a black solid and precipitates gradually. The RGO is then repeatedly rinsed with water and ethanol and filtered, then air dried to get the solid product. The exfoliated GpO or GO in water exhibits hydrophilic behavior due to the oxygen contents as mentioned previously, but the RGO aggregates together due to the fact that the chemical linkages inside the interlayers are removed, thus the RGO sheets become highly hydrophobic and shrink together. More importantly, the structural characterization can be verified by Raman microscopy for the first-order scattering of E_2_g mode (G) and the extensive oxidation in-plane sp^2^-domains. It should be pointed out that not only the hydrazine hydrate is used in the reduction of GO, but the number of reducing agents known to date has increased, as well. The reducing agents can be categorized for creating good supports and for doping purposes. For instance, borohydrides, aluminium, hydride and hydrohalic acid are reducing agents which are used for good-support purposes while hydrazine, isopropanol, and l-ascorbic acid are used for doping purposes. The list of these reducing agents for chemical reduction of graphite oxide is also discussed elsewhere [31].

Regarding the chemical reduction of GO, the advantages of this method are as follows: (1) the technique is a cost-effective and simple process using cheap chemical solutions; (2) the method provides a high yield of graphene dispersion scalable for industry; (3) the chemically-derived graphene forms highly stable colloids; and (4) large-size graphene layers can be produced and this facilitates macroscopic fabrication.

### 2.3. Graphene-Based Quantum Dots

This new material is called “carbon-based dots”. The discovery of carbon-based dots happened accidentally during purification of single-walled carbon nanotubes (SWNTs) from arc-discharge soot. The fluorescent carbon obtained had different color emissions under UV light when several sizes and concentrations of chopped SWNT dispersions were irradiated. These carbon particles were subsequently called carbon quantum dots (CQDs) [89]. For consistency, the word “dot” involving “quantum” is distinguished from the spherical semiconductors. The dots are obtained due to the size reduction of basal-plane carbon structures to a few nanometers approaching that of the dots with quantum phenomena. More importantly, the new materials are less toxic and are compatible for cellular internalization as verified by many groups [17,24,90,91,92].

**Figure 5:**
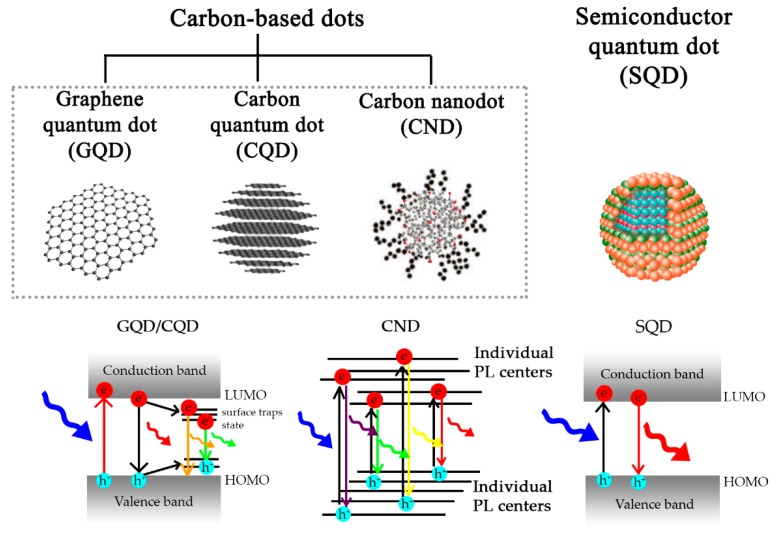
Schematic illustration and photoluminescent mechanism of carbon-based dots, including graphene quantum dot (GQD), carbon quantum dot (CQD), carbon nanodot (CND), compared to the quantum dots made of semiconductor (SQD).

Figure 5 shows the photoluminescent carbon-based dots categorized into GQD, CQD, and CND as differentiated to the SQD [93]. Reed [94] has defined the term of the SQD of sizes in the quantum-confined regime which is less than the Bohr exciton radius, typically within a few nanometers. The fine SQDs display resonant tunneling with discrete density of states. As explained, the carbon-based dots are based on structures with sp^2^-sp^3^ hybridized domains of carbon materials in which the structure of graphene exists as a core. Moreover, the term quantum dot is also reserved for the carbon-based dots that satisfy the nanometer-size and functionalized structures with the quantum confinement.

The GQDs can be made up of a single atom of graphite layer or graphene chopped into fragments in a range of 2–20 nm. The disk feature of GQDs is very tiny, analogous to the dot [24,95]. Upon either stimulus by UV light or electrical energy, the excitons would be confined to the quantized energy levels and release photoluminescent light in a narrow band. Interestingly, the major material precursors are not only the infinite graphene sheet and the functionalized graphene, but some organic molecules are also applicable. For the top-down methods the hydrothermal cutting produces several nanosized sheets while the bottom-up methods use direct pyrolysis and carbonization of the organic precusors.

The nano- to micro-sized fragments of GO from hydrothermal cutting were passivated by placing -C=O, -OH and -COOH groups on the surface [96]. Suvarnaphaet [24] achieved high quantum yield of the GO-like structure from limeade and applied it for rapid sensing of heavy-metal ions, for bioimaging, and it was tested for biocompatibility and found to be non-toxic to human kidney cells. Dong [97] also found nano-sized GO−like structures synthesized from humic substances in the presence of nitrogen-containing groups such as -NH_2_ and C=N on the surface of the carbon-based dots causing an enhancement in quantum yield. Natural and artificial aromatic carbon substances have also been investigated enabling a fine tuning of crucial optical properties, such as tunable photoluminescence. These aromatic carbon substances can be employed as effective labeling molecules as recently reported, for example, polycyclic aromatic hydrocarbon (PAH) [98], chloroform (CHCl_3_) and diethylamine (DEA) [99], orange juice [100], potato [101], citric acid and linear-structured polyethyleneimine (LPEI) polymer [102]. The multicolor emission spectra were due to the different sizes and nature of the surface functional groups, i.e., C=O and C=N. To obtain controllable quantum effects the size can be reduced down to a few nanometers, and the carbon core structure can be chemically modified. Thus the as-prepared carbon-based dots always contain sp^2^- and sp^3^- hybridized structures leading them to be modified by the oxygen/nitrogen functionalized groups and protein modification. The fluorescence properties and their mechanism arises from the localization of electron-hole (e-h) pairs which form excitons analogous to conventional QDs. The conjugated π-domains in the carbon-core structure arising from modifying surface chemistries and defects are also additionally promoted [93]. The strategies for chemical synthesis are used to stabilized the products be a probe for specific applications in in vivo bioimaging and biomarkers [24,90,95], to improved drug delivery [103,104,105], and to be integrated for biosensors [21].

## 3. Engineering of Biosesnsor Devices Using Graphene-Based Materials and Current Progress

**Figure 6:**
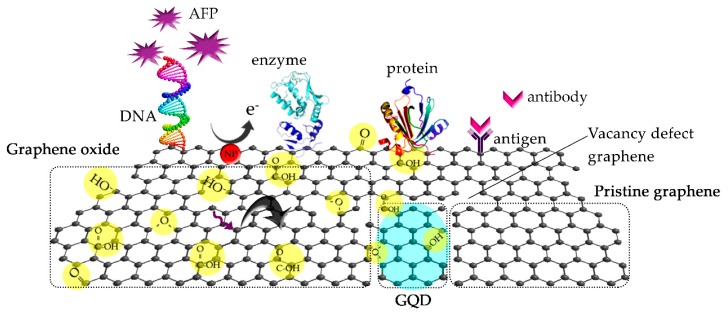
Schematic illustration of the graphene-based materials that can be immobilized with biomolecules as the receptor.

Previously, the structures and outstanding properties of graphene-based materials using different synthesis methods have been exploited in biosensing applications since graphene is a semi-metal with ultra-high charge mobility giving excellent electronic properties, having large surface area, being capable of being functionalized on its surface. There are many possible approaches to engineer the receptor for targeting biomolecules. In the biomedical field, pristine graphene is not only referred to as an oxide-free graphene presenting π-π stacking, non-covalent interactions and high electrostatic force, but it also offers an infinite surface at a molecular level. Therefore, graphene provides for a high possibility of active sites for charge-biomolecular interactions due to the large specific surface area leading to a sensing enhancement as well as supporting the desired functionalization to target biomolecules to improve the selectivity. Figure 6 illustrates the points of view of the possible interactions of the graphene-based material system. For example, the pure graphene area as shown in the figure can provide a charged area to absorb any charged molecules or metal ions as well as interactions at a vacancy defect. The functionalized graphene area is able to directly detect the biomolecules by its own oxide components due to the synthesis in which lots of epoxide, hydroxyl and carboxyl groups are formed on the edge and surface sites. In addition, the functionalized graphene allows binding of heteroatoms, nanoparticles (NPS), quantum dots (QDs), DNA, enzymes, proteins, antigens, antibodies, and other specific molecules [26,106,107].

In biomedical applications, graphene-based nanostructures have been recently reported with highly sensitive and selective performance as biosensors as listed in Table 1.

### 3.1. Engineering of Pristine Graphene−Biomolecule- based Biosensors

**Figure 7:**
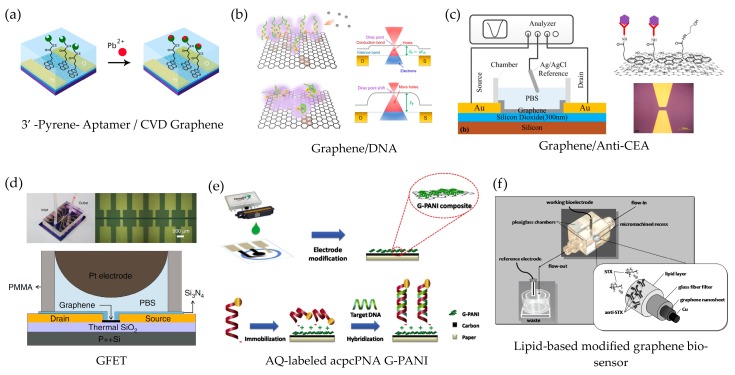
Schematic illustrations of graphene-based biosensors: (**a**) Pb^2+^ in blood biosensor based on GFET (reprinted with permission from [49]); (**b**) Pb^2+^ biosensor based on graphene/DNA (reprinted with permission from [109]); (**c**) CEA protein biosensor based on graphene/anti-CEA (reprinted with permission from [108]); (**d**) real-time binding kinetics and affinity of DNA hybridization based on GFET (reprinted with permission from [50]); (**e**) paper-based biosensor for human papillomavirus (HPV) detection (reprinted with permission from [127]); and (**f**) a lipid-based modified graphene electrochemical biosensor (reprinted with permission from [135]).

In the graphene-based biosensors, graphene is able to enhance the sensitivity and LOD as well as the performance of the biosensor device by improving the charge or electron transfer between graphene and the biomolecules due to its extraordinary properties. For example, as seen in Figure 7, a label-free and portable aptasensor utilizes pristine graphene as the electrode in a field-effect transistor device [49]. The GFET biosensor is used to detect the Pb^2+^ ions in children’s blood, in which the blood matrix is very complicated. The mechanism to distinguish Pb^2+^ ions from common ions in the blood, including Na^+^, K^+^, Mg^2+^ and Ca^2+^ at lower 0.1 M/L, is intrinsically p-doping on the CVD graphene and the surface engineering by G-quadruplex, Thrombin binding aptamer (TBA), and 8–17 DNAzyme. The lowest concentration of GFET aptasensor is at 37.5 ng/L, which was approximately one thousandth of the safety limit (100 μg/L) for Pb^2+^ in blood. A GFET construction shows a similar Pb^2+^ detecting platform but using G-quadruplex as the receptor [109]. The mechanism is due to the electrostatic potential change after the lead combines to the double layer of DNA/CVD graphene electrodes leading to the shift in Dirac point in the band structure of graphene. For this device, the LOD is only 163.7 ng/L for the first signal verification of DNA/GFET. Carcinoembryonic antigen (CEA) is a protein that can be measured in the blood of a cancer patient. Recently, a label-free immunosensor based on the antibody-modified graphene FET was reported [108]. The surface modification is applied via a non-covalent functionalization and π-stacking using a pyrene and a reactive succinimide ester group to interact with graphene. The GFET biosensor shows the specific monitoring of the CEA protein in real-time with high sensitivity of <100 pg/mL. In the precise quantitative measurement of DNA concentrations as well as binding affinities and kinetics of DNA hybridization, an array of six CVD graphene-based FETs was fabricated in a single multiplexed sensor for DNA analysis [50]. The concentration of oligonucleotides can be measured as low as 10 pM in which the single-single-base mutations can be analyzed in real time.

In the use of electrochemical properties of graphene material, a novel paper-based biosensor for human papillomavirus (HPV) detection was reported [127]. The graphene-polyaniline (G-PANI) electrode is modified using an anthraquinone-labeled pyrrolidinyl peptide nucleic acid (acpcPNA) probe (AQ-PNA) and printed by inkjet printing method. In a presence of surface engineering of a negatively charged amino acid on graphene electrode through the electrostatic attraction, a synthetic 14-base oligonucleotide target with a sequence corresponding to human papillomavirus (HPV) type 16 DNA is measured the electrochemical signal response of the AQ label to identify the primary stages of cervical cancer.

On the development of electrochemical technology, graphene microelectrodes integrated with bilayer lipid membranes (BLMs) have shown promising results in both static and stirred experiments [135]. Moreover, due to the support made of lipid film, the biosensor achieves a good reproducibility, reusability, high selectivity, rapid response times, long-shelf life, and high sensitivity. This enables a direct potentiometric measurement. Nikolelis et al. have also reported the use of the graphene microelectrodes in detecting toxicants, i.e., carbofuran in fruit [132], saxitoxin [136], cholera toxin [137] and for diagnosis of d-dimers [133], urea [138] and cholesterol as seen in Figure 7f.

### 3.2. Engineering of Biomolecules-Functionalized Graphene Based Biosensors

**Figure 8:**
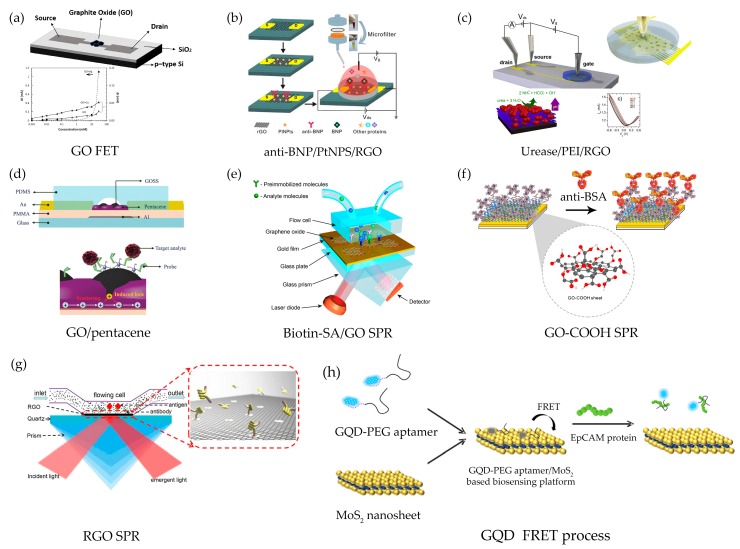
Schematic illustration of functionalized graphene-based biosensors: (**a**) a glucose detection based on GO FET (reprinted with permission from [111]); (**b**) DNA detection based on printing GO/pentacene FET (reprinted with permission from [110]); (**c**) urea platform biosensor based on Urease/PEI/RGO FET (reprinted with permission from [112]); (**d**) Heart failure detection based on Pt NPS/RGO FET (reprinted with permission from [113]); (**e**) Biotin-SA/GO SPR chip (reprinted with permission from [114]); (**f**) BSA biosensor based on GO-COOH enhanced SPR (reprinted with permission from [117]); (**g**) rabbit IgG detection based on RGO SPR (reprinted with permission from [116]); and (**h**) FRET biosensor based on GQD-PEG aptamer/MoS_2_ (reprinted with permission from [23]).

Biosensors based on the subtype of graphene materials or functionalized graphene GO, RGO, and GQD are widely used in medicine, biomedical, and bioimaging regimes. This is due to their extremely large surface area and ability to interact with various types of molecules. In addition, the outstanding properties of solubility, biocompatibility, and functionalization play an important role in sensing mechanisms. Currently, as shown in Figure 8, the GO-based FET has simply demonstrated glucose detection without an enzymatic glucose solution [111]. In this device, GO is used as the selective material to glucose while the sensitivity of the sensor is enhanced down to 1 μM by adding CuNPS and AgNPs. Interestingly, functionalized graphene oxide (GO) ink has been printed on a pentacene FET for detecting artificial DNA and circulating tumor cells [110]. Upon capturing the DNA by its phosphate group, the negative charge attracts holes at the grain-boundary of the pentacene layer and induces the collision or scattering in the region of the pentacene layer. Therefore, the mobility of the FET changes extremely achieving a high sensitivity of 0.1 pM and this could be improved for mass-scale production of printed biosensors. In a platform for ultra-sensitive urea detection, the RGO surface is decorated by the new construction of layer-by-layer assemblies of polyethylenimine (PEI) and urease [112]. The RGO FET of urea detection can be analyzed as the change of pH in liquid gate, by which shifting the Dirac point at the minimum voltage of <500 mV. The limit of urea detection is down to 1 μM with very fast response and good long-term stability. In addition, the introduction the Cu^2+^ improves the LOD down to 0.01 μM. In recent complex platforms, FET biosensors using RGO combined with PtNPS and anti-BNP have been explored as a brain natriuretic peptide (BNP) detector at the early stage level [113]. The BNP is a recognized biomarker and it is very important in heart failure diagnosis and prognosis. The RGO FET achieves the lowest of detection at 100 fM in a human whole blood sample.

Surface plasmon resonance (SPR) is a widely used technique to investigate biochemical reactions in scientific research and medical diagnosis [139]. In particular, SPR provides label-free biosensing and real-time monitoring of biomolecule interactions. However, for small molecules or at low concentrations of the targets, the SPR signal is not sufficient to be analyzed. To improve the SPR signal and biosensing performance, linking layers of GO have been introduced into the SPR sensor system [114]. The sensor chip consists of gold sensor chips using PMMA as an intermediate membrane, s monolayer of CVD graphene on top, and the biotin-SA conjugate, respectively. The SA molecules allow the biosensor to select the immobilized biomolecules containing biotin. The linking layers of GO in the system provides a number of binding sites for biomolecules due to the large surface area. However, a GO thickness of more than 10 nm strongly limited the optical absorption leading to a sensitivity reduction. Recently, there has been a demonstration showing the improvement and control of the plasmonic coupling mechanism in GO SPR-based immunoaffinity biosensors by adding carboxyl groups [117]. The GO-COOH SPR chip can be improved four times over the SPR angle shift and achieved the lowest antibody detection limit of 0.01 pg/mL. Other work has reported the reduction of GO-based SPR fabricated by thermal reduction at high temperature, the so-called RGO SPR, with a thickness of 8.1 nm [116]. The performance of the RGO SPR biosensor shows a response to rabbit immunoglobulin G (rabbit IgG) with a LOD of 0.0625 μg/mL.

Currently, fluorescence biosensors based on the GQDs have gained much attention as an alternative choice due to their ease of the synthesis, good stability, fast tissue internalization, and biocompatibility. The fluorescence biosensor relies on the energy transfer between the electron donor and acceptor which is powerful for drug delivery and biomolecular interactions at the nanoscale. Fluorescence resonance energy transfer (FRET) is a mechanism to describe the energy transfer between two fluorescent molecules, where one is a donor being in an excited state and ready to transfer to the other one or an acceptor via a non-radiative dipole-dipole coupling [140].

This simple technique has been reported in a novel FRET based on GQD-PEG aptamer/MoS_2_ for the detection of epithelial cell adhesion molecule (EpCAM), which is a glycosylated membrane protein expressed on the surface of circulating tumor cells (CTCs) [23]. In the mechanism, GQD is used as the FRET donor that emits fluorescence at 466 nm under an excitation of 360 nm. MoS_2_ having a good quenching ability is the acceptor. When the PEG is conjugated onto GQD, the PEGylated/GQD exhibits a stronger fluorescence emission because of the quantum confinement. Then the PEGylated/GQD is conjugated with the aptamer via the van der Waals binding causing a proximity of GQD and MoS_2_ and quenching GQD. When the EpCAM protein with a strong binding affinity is introduced, the GQD could label on EpCAM aptamer and restores its fluorescence. Therefore, the EpCAM target protein can be monitored by the fluorescence emission.

## 4. Conclusions

In this review, we discuss our points of view on the intrinsic properties of graphene and its surface functionalization concerned with the transduction mechanisms in biomedical applications. We also explain several well-known techniques used for the synthesis of graphene-based materials and their properties. A variety of graphene-based materials have been made consisting of pristine graphene and the functionalization of graphene oxide, reduced graphene oxide and graphene quantum dot. The mechanisms are discussed with respect to the most recent biosensing devices for drug delivery, biosensors, healthcare sensors, bioimaging, and other novel techniques.

Graphene is one of the most well-known 2D materials. The major characteristic and properties of graphene are outstanding, i.e., zero-bandgap semiconductor, linear-like at the Dirac point, relativistic-like charge velocity, ultra-high charge mobility, transparency, large surface area, non-toxicity, having proximity induced ability, high tensile strength and high thermal conductivity, etc. However, to maintain those properties, graphene has to be perfectly proper. Alternative techniques by chemical synthesis that serve for ambient circumstances are also presented. In the synthetic procedures, the graphite is modified and functionalized by various oxygen-containing groups. In addition, the functionalized graphene is always contaminated by impurities, defects, and disorder. Hence, the structure of graphene would be importantly changed, especially the electronic properties will be distorted. On the other hand, the presence of the oxygen-containing groups and the ability to functionalize onto the graphene-based structure are important in the electrochemistry of the biosensing applications such as for labeling biomolecule recognition, for enhancing the sensing signal, for increasing the number of active sites and active area, and for probing biomolecules in an imaging application. A novel variety of the graphene-based biosensors is presented in the last section. The engineering of biosensing platforms, the mechanisms and techniques are discussed. Many new approaches are discussed in detail in this review.

Since graphene material has been very well established, other 2D materials have now been explored. This opens up a wide range of possibilities and plays a crucial role in sensor and biosensor applications utilizing the largest surface area. In the upcoming future, these 2D novel materials will be further developed and tailored for specificity of bioreceptors. These materials can be employed and integrated in different sensor and biosensor platforms giving an ultra-high sensitivity and may provide a solution to some challenges, such as early stage cancer detection.

## Figures and Tables

**Table 1:** Current generation reports of graphene-based biosensors.

Technique	Receptor System	Target Biomolecules	Limit of Detection	References
FRET ^1^	Boron-doped GQDs ^2^/ATP ^3^	Ce^3+^ ions in MCF-7 ^4^ cells	0.4 mM in 10 ± 5 cell/mL	[21]
FRET	MWCNTs@GONRs ^5^	dual tDNAs (P35s ^6^ and TNOS ^7^)	0.35 nM for P35 s 0.5 nM for TNOS	[22]
FRET	GQD-PEG-aptamer/MoS_2_	EpCAM ^8^	450 pM	[23]
GFET ^9^	Graphene/Tris-HCl	Pb^2+^	<37.5 ng/L	[49]
GFET	Graphene/Anti-CEA ^10^	CEA protein	<100 pg/mL	[108]
GFET	Graphene/DNA	Pb^2+^	163.7 ng/L	[109]
GFET	Graphene	DNA	10 pM	[50]
GO ^11^ FET	GO/pentacene	Artificial DNA	0.1 pM	[110]
GpO ^12^ FET	GpO/Cu or AgNPS	Glucose	1 μM	[111]
RGO ^13^ FET	Urease/PEI ^14^/RGO	Urea	1 μM	[112]
RGO FET	PtNPS	BNP ^15^	0.1 pM	[113]
GSPR ^16^	Biotin-SA ^17^/GO	DNA	-	[114]
GLSPR ^18^	Ni/graphene	3-NT ^19^	0.13 pg/mL	[115]
SPR	RGO	Rabbit IgG ^20^	0.3125 μg/L	[116]
SPR	Au/GO–COOH	Anti-BSA ^21^	0.01 pg/mL	[117]
SPR	*M. lysodeikticus*/GO	Lysozyme in serum	0.05 μg/mL	[118]
SPR	GO/(N-) PPLRINRHILTR(-C) ^22^	HCG ^23^	0.065 nM	[119]
Fiber optic SPR	Ag-MoS_2_-Graphene	DNA	1 μM	[120]
SPR	Graphene–MoS_2_	ssDNA	-	[121]
ECHEM ^24^	AuNPS/GO	MCF-7	0.0375 μg/mL	[122]
ECHEM	NH_2_-GS/Au@Pt/PDA-N-MWCNT ^25^	AFP ^26^	0.1 pg/mL	[123]
ECHEM	FAO ^27^/N-doped graphene/AuNPS/FTO	HbA1c ^28^	0.2 μg/mL	[124]
ECHEM	Pd-Au@carbon dots	*Colitoxin* DNA in human serum	1.82 × 10^−17^ M	[125]
ECHEM	Ni-MG-BDD ^29^	Glucose	0.24 μM	[126]
ECHEM	AQ-labeled acpcPNA ^30^ G-PANI	HPV-DNA type 16	2.3 nM	[127]
ECHEM	GO-ssDNA/Au	VEGF ^32^	0.05 ng/mL	[128]
	PLLA ^31^/GO-ssDNA/Au	PSA ^33^	1 ng/mL	
ECHEM	MoS_2_-Graphene/L-cysteine	PTH ^34^	1 pg/mL	[129]
ECHEM	MoS_2_/graphene	ctDNA ^35^	0.0001 pM	[130]
ECHEM	AuNPS/MoS_2_/graphene/GCE ^36^	DNA	0.0022 pM	[131]
ECHEM	Calix[4]arene phosphoryl/graphene electrode	Carbofuran	1 μM	[132]
ECHEM	Anti human d-dimer antibody/lipid film/graphene nanosheets	d-dimer	1 μM	[133]
Electron transfer	MoS_2_/GO	Glucose in human serum	65 nM	[134]

*Notes*: ^1^ FRET: fluorescence resonance energy transfer, ^2^ GQDs: graphene quantum dots, ^3^ ATP: adenosine triphosphate, ^4^ MCF-7: Michigan Cancer Foundation-7 (breast cancer cells),^5^ GONRs: graphene oxide nanoribbons, ^6^ P35s: promoter cauliflower mosaic virus 35 s, ^7^ TNOS: terminator nopaline synthase (from transgenic soybean), ^8^ EpCAM: epithelial cell adhesion molecule, ^9^ GFET: graphene field effect transistor, ^10^ CEA: carcinoembryonic antigen, ^11^ GO: graphene oxide, ^12^ GpO: graphite oxide, ^13^ RGO: reduced graphene oxide, ^14^ PEI: polyethylenimine, ^15^ BNP: brain natriuretic peptide (heart failure), ^16^ GSPR: graphene based surface plasmon resonance, ^17^ Biotin-SA: biotin-streptavidin, ^18^ GLSPR: graphene localized surface plasmon resonance, ^19^ 3-NT: 3-nitro-l-tyrosine, ^20^ Rabbit immunoglobulin G, ^21^ BSA: bovine serum albumin protein, ^22^ (N-) PPLRINRHILTR(-C): (N-Pro-ProLeu-Arg-Ile-Asn-Arg-His-Ile-Leu-Thr-Arg-C), ^23^ HCG: human chorionic gonadotropin protein, ^24^ ECHEM: electrochemistry, ^25^ PDA-N-MWCNT: poly-dopamine functionalized N-doped multi-walled carbon nanotube, ^26^ AFP: Alpha fetoprotein, ^27^ FAO: fructosyl amino-acid oxidase, ^28^ HbA1c: Glycatedhemoglobin, ^29^ Ni-MG-BDD: nickel-microcrystalline graphite-boron doped diamond, ^30^ acpcPNA: anthraquinone-labeled pyrrolidinyl peptide nucleic acid, ^31^ VEGF : vascular endothelial growth factor, ^32^ PLLA: poly-L-lactide, ^33^ PSA: prostate-specific antigen, ^34^ PTH: parathyroid hormone, ^35^ ctDNA: circulating tumor DNA, ^36^ GCE: carbon glassy electrode.

## Abbreviations

The following abbreviations are used in this manuscript:

GO	Graphene oxide
GpO	Graphite oxide
RGO	Reduced graphene oxide
GQD	Graphene quantum dot
SWNT	Single-walled carbon nanotubes
2D	Two-dimensional
LOD	Limit of detection
HOPG	Highly oriented pyrolytic graphite
FET	Field-effect transistor
GFET	Graphene field-effect transistor
CVD	Chemical vapor deposition
SiC	Silicon carbide
NMP	*N*-Methylpyrrolidone
DMA	*N*,*N*-Dimethylacetamide
GBL	alpha-butyrolactone
DMEU	1,3-Dimethyl-2-imidazolidinone
UHV	Ultrahigh vacuum
EG	Epitaxial graphene
FTIR	Fourier transform spectroscopy
ESR	Electron spin resonance
XPS	X-ray photoemission spectroscopy
SQD	Semiconductor quantum dot
FRET	Fluorescence resonance energy transfer
GQDs	Graphene quantum dots
ATP	Adenosine triphosphate
MCF-7	Michigan Cancer Foundation-7
GONRs	Graphene oxide nanoribbons
P35s	Promoter cauliflower mosaic virus 35 s
TNOS	Terminator nopaline synthase
EpCAM	Epithelial cell adhesion molecule
GFET	Graphene field effect transistor
CEA	Carcinoembryonic antigen
PEI	Polyethylenimine
BNP	Brain natriuretic peptide
GSPR	Graphene based surface plasmon resonance
Biotin-SA	Biotin-streptavidin
GLSPR	Graphene localized surface plasmon resonance
3-NT	3-Nitro-l-tyrosine
Rabbit IgG	Rabbit immunoglobulin G
BSA	bovine serum albumin protein
(N-)PPLRINRHILTR(-C)	(N-Pro-ProLeu-Arg-Ile-Asn-Arg-His-Ile-Leu-Thr-Arg-C)
HCG	Human chorionic gonadotropin protein
ECHEM	Electrochemistry
PDA-N-MWCNT	Polydopamine functionalized N-doped multi-walled carbon nanotube
AFP	Alpha fetoprotein
FAO	Fructosyl amino-acid oxidase
HbA1c	Glycated hemoglobin
Ni-MG-BDD	Nickel-microcrystalline graphite-boron doped diamond
acpcPNA	Anthraquinone-labeled pyrrolidinyl peptide nucleic acid
VEGF	Vascular endothelial growth factor
PLLANPs	Poly-l-lactide nanoparticles
PSA	Prostate-specific antigen
PTH	Parathyroid hormone
ctDNA	Circulating tumor DNA
GCE	Carbon glassy electrode
TBA	Thrombin binding aptamer
